# HPClas: A data‐driven approach for identifying halophilic proteins based on catBoost

**DOI:** 10.1002/mlf2.12125

**Published:** 2024-07-20

**Authors:** Shantong Hu, Xiaoyu Wang, Zhikang Wang, Menghan Jiang, Shihui Wang, Wenya Wang, Jiangning Song, Guimin Zhang

**Affiliations:** ^1^ College of Life Science and Technology Beijing University of Chemical Technology Beijing China; ^2^ Monash Biomedicine Discovery Institute and Department of Biochemistry and Molecular Biology Monash University Melbourne Victoria Australia

**Keywords:** feature engineering, halophilic protein, machine learning

## Abstract

Halophilic proteins possess unique structural properties and show high stability under extreme conditions. This distinct characteristic makes them invaluable for application in various aspects such as bioenergy, pharmaceuticals, environmental clean‐up, and energy production. Generally, halophilic proteins are discovered and characterized through labor‐intensive and time‐consuming wet lab experiments. In this study, we introduce the Halophilic Protein Classifier (HPClas), a machine learning‐based classifier developed using the catBoost ensemble learning technique to identify halophilic proteins. Extensive in silico calculations were conducted on a large public dataset of 12,574 samples and HPClas achieved an area under the receiver operating characteristic curve (AUROC) of 0.844 on an independent test set of 200 samples. The source code and curated dataset of HPClas are publicly available at https://github.com/Showmake2/HPClas. In conclusion, HPClas can be explored as a promising tool to aid in the identification of halophilic proteins and accelerate their application in different fields.

## Impact statement

A method based on prediction of proteins secreted by extreme halophilic bacteria was used to successfully extract a large number of halophilic proteins. Using these data, we have trained an accurate halophilic protein classifier that could determine whether an input protein is halophilic with a high accuracy of 84.5%. This work could not only promote the exploration and mining of halophilic proteins in nature but also provide guidance for the generation of mutant halophilic enzymes.

## INTRODUCTION

Since halophilic microorganisms (halophiles) are adapted to extreme environments, they are conducive to pollution‐free productions that do not require sterilization^1^. In addition, their proteins, which show a strong dependency on high‐salt conditions^2^, are referred to as halophilic proteins. Halophilic proteins commonly require high NaCl concentrations to be active, while halophilic enzymes depend on salt for their catalytic activity. For example, the α‐amylase from *Halothermothnx orenii* shows high activity at 4.7 mol/l NaCl^3^. Moreover, in‐depth studies have found that some structural elements in halophilic enzymes, originally adapted for high‐salt conditions, can also contribute to ensure their tolerance to organic solvents^4^, so that they have wide applications in anhydrous environments. Moreover, halophilic enzymes also show broad substrate specificity. These unique properties render them potentially valuable in various fields such as biofuel production, textile processing, waste treatment, and detergent additives^5^. Halophilic proteins can also serve as crucial enzymes in building metabolic pathways throughout the production process^1^.

Given the wide range of potential applications of halophilic proteins, their identification has become a major area of research interest. Many studies have collected samples of halophilic bacteria and archaea from salt lakes and oceans worldwide, using whole‐genome sequencing and wet laboratory validation methods to screen for halophilic proteins. Indeed, advances in DNA sequencing technologies, particularly the utilization of genomics and metagenomics tools, have facilitated the discovery of large numbers of protein sequences from a diverse spectrum of organisms. In 2014, Sharma et al. established a halophilic protein database HProtDB^6^, and researchers noticed that although most proteins contained in this database come from halophilic bacteria, proteins derived from halophilic bacteria may not be halophilic proteins. Some halophilic bacteria, such as *Halobacterium*, can effectively regulate their intracellular salt concentrations to be lower than the extracellular environment^7^. They can also synthesize and accumulate compounds known as compatible solutes (e.g., proline, lysine, and ectoine) to balance osmotic pressure inside and outside the cell. Therefore, these bacteria may contain some nonhalophilic proteins. The current paradigm for halophilic protein identification relies on laboratory experiments to assess the halophilic characteristics of proteins. Nevertheless, this approach is quite inefficient and costly, and cannot meet the growing demand. Therefore, computational methods for halophilic protein screening are expected to address this issue and have attracted increasing research attention. However, previous methods suffered from severe data scarcity and performance generalization issues^8^, ^9^. Furthermore, the lack of user‐friendly software or websites hinders direct access by the public. Hence, there is a need for a powerful tool with greater accuracy that is readily available to the public to help unlock the potential of halophilic proteins.

**Figure 1:**
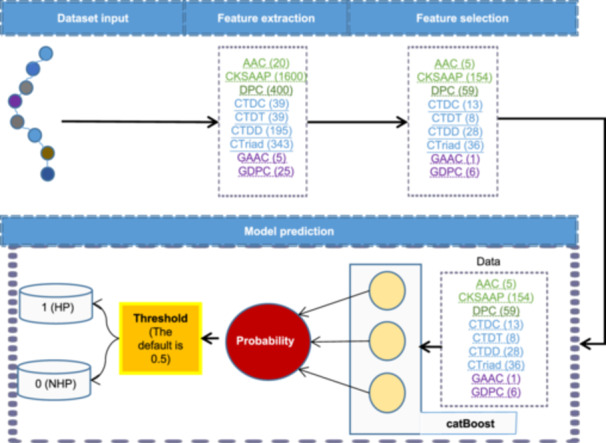
Overall architecture of Halophilic Protein Classifier (HPClas). The input to HPClas is a protein sequence without a signal peptide. In the feature extraction process, features were extracted through feature descriptors, and feature selection was used to optimize the model. HP, halophilic protein; NHP, nonhalophilic protein.

In this study, we introduced a novel data collection method and created a large and comprehensive dataset of halophilic proteins. On this basis, we reported a catBoost‐based machine learning (ML) model termed Halophilic Protein Classifier (HPClas) for the identification of halophilic proteins (Figure 1). HPClas was trained using high‐quality data from UniProtKB and the NCBI protein database. This model takes amino acid sequences as inputs and is capable of predicting whether the protein is halophilic. We compared the performance of HPClas with state‐of‐the‐art (SOTA) models on the test set of a previous study. Experimental results demonstrated that HPClas outperformed existing halophilic protein prediction tools. Furthermore, we also conducted an interpretation analysis of our model, which further reinforced the credibility of our predictions.

## RESULTS AND DISCUSSION

### Collection of a large training dataset of halophilic and nonhalophilic proteins

In previous studies^8^, ^9^, halophilic and nonhalophilic protein datasets were constructed but not made public, and all proteins in the genome of *Salinibacter ruber* DSM 13855 have been proven to be halophilic, whereas all proteins in the *Pelodictyo luteolum* DSM 2379 have been confirmed to be nonhalophilic. We downloaded these proteins from UniProtKB^10^ as a part of the training dataset (Table 1).

**Table 1:** Overview of the training dataset and independent test.

Application	Label	Source	Key words	Quantity before cleaning	Quantity after cleaning
Training	Positives	NCBI‐protein	Halophilic bacteria	445,472	7462
			Halophilic archaea	444,825	
			*Salinibacter ruber* DSM 13855	6106	
	Negatives		*Anabaena* (fresh water)	334,599	5112
			*Beggiatoa* (fresh water)	41,601	
			*Flectobacillus* (fresh water)	36,623	
			*Limnothrix* (fresh water)	29,423	
			*Vibrio cholerae* (fresh water)	37,021	
			*Pelodictyo luteolum* DSM 2379	4537	
Testing	Positives	UniProt	Halophilic bacteria	357,117	100
	Negatives		*Chryseobacterium salivictor* NBC 122	2837	100

Additionally, here, a novel method is proposed to generate a more comprehensive dataset of halophilic proteins. This method involves predicting secreted protein from halophilic microorganisms, enabling the identification of secreted proteins. These proteins are expected to adapt to high‐salt conditions, and thus categorized as halophilic proteins^11^. The comprehensive dataset contains a training set and an independent test set from the NCBI Protein Database and UniProtKB. Detailed information such as the application, labels, sources, key words, and quantities before and after cleaning of the data set is summarized in Table 1. The training set contains 7462 halophilic proteins and 5112 nonhalophilic proteins, while the test set contains 100 pairs of halophilic and nonhalophilic proteins.

Wet experimentally validated halophilic proteins are extremely limited and challenging to retrieve, making the collection of a comprehensive dataset quite difficult. However, Zhang et al.^8^, ^9^ have developed two classification models for halophilic proteins. For both models, the positive samples were from the halophilic bacterium, *S. ruber* DSM 13855^12^, which did not rely on proton pumps to regulate salt concentration, ensuring that all proteins from this bacterium were halophilic proteins. Based on previous studies, we proposed a method to predict halophilic proteins by screening secreted proteins in halophilic bacteria. Secreted proteins that evolved in high‐salt environments often function extracellularly and tend to show a preference for salt tolerance. This strategy ensured the high quality and quantity of the current dataset, while strict steps for removing redundancy including the removal of highly homologous sequences and the imposition of length criteria further reduced the risk of data leakage and ensured data quality. Moreover, proteins from multiple bacterial species increased the diversity of the data compared to proteins from a single strain source.

During the data mining process, not only did the internal homogeneity within the independent test set fall below 40%, but also the homogeneity between the entire independent test set and the training set was less than 25% to ensure a substantial distributional difference from the training set. Such high‐quality data facilitate further assessment of the model's generalization, and thus, we chose it as a benchmark for optimizing the model.

Of course, our data have certain limitations. For example, the data sources are mainly secreted proteins, which may lead to potential misjudgments when predicting cytoplasmic proteins. To alleviate these limitations, we attempt to enrich the training dataset. For instance, the entire proteome of strain *S. ruber* DSM 13855 was used as a positive sample and strain *P. luteolum* DSM 2379 was used as a negative sample. We hope to have more experimental data in the future that will allow us to obtain more intracellular protein data, so that we can further optimize our model.

### Combination screening of models and feature encoding methods

Eleven feature descriptors were used to encode protein sequences into biologically meaningful vector representations, where each descriptor represents a statistical perspective on amino acid sequences. After feature extraction, we found that some feature values varied a lot, with some ranging from 0 to 0.01 and some from 1 to 1000. These nonnormalized features might considerably hinder model converge and affect network performance. Therefore, we used the MinMaxScaler method to normalize the values of different features, ensuring that all values fell within the range of 0 to 1. The results are presented at https://github.com/Showmake2/HPClas/features.

To assess the contribution of different descriptors to halophilic protein predictions, we initially adopted an iterative approach to systematically exclude individual descriptors from three different models including catBoost^13^, XGBoost^14^, and Random Forest (RF)^15^. Since the dataset is imbalanced, the precision is insufficient to correctly evaluate the performance of the model; therefore, AUROC^16^ (area under the receiver operating characteristic curve) and AUPRC^17^ (area under the precision–recall curve) were used as the main evaluation matrix. We then compared the difference in AUROC and AUPRC between models trained with the full descriptor set and models that omitted specific descriptors. Furthermore, we calculated the AUROC value and the AUPRC value of each descriptor when trained individually, thereby quantifying the importance of each descriptor.

To illustrate feature importance analysis, we could take the amino acid composition (AAC) descriptor as an example. The calculated formula of its importance score is as follows: AAC importance (AUPRC) = [AUPRC (all features)–AUPRC (all‐AAC)] × 10,000. This essentially quantified the extent to which model performance degrades when AAC is removed from the full feature set. A positive importance score indicates that AAC improves the model, while a negative score means that it worsens performance. We applied this methodology to calculate the importance of each descriptor for each ML model separately. For a given model, if a descriptor had a positive importance score, besides its individual AUROC and AUPRC exceeding 0.6, we would consider that it had contributed positively to the model. Descriptors that do not meet these criteria were deemed to have a negative contribution.

**Figure 2:**
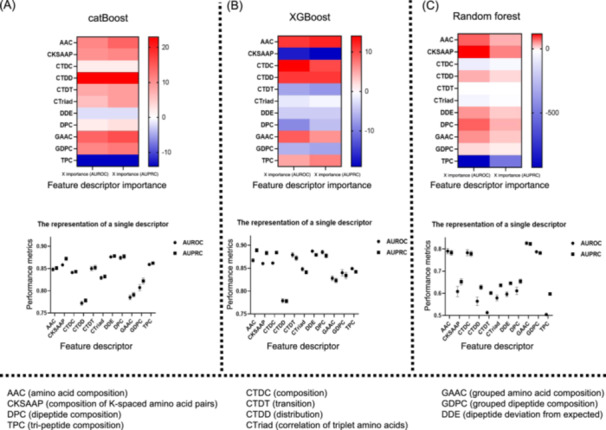
Importance and performance comparison of each descriptor (AAC, CKSAAP, DPC, and so forth) for different machine learning models on fivefold cross‐validation. The detailed explanations of these descriptors are provided in Material and Methods Section. (A) Importance and performance of feature descriptors based on catBoost. (B) Importance and performance of feature descriptors based on XGBoost. (C) Importance and performance of feature descriptors based on random forest (RF). AUPRC, area under the precision–recall curve; AUROC, area under the receiver operating characteristic curve.

To evaluate the contribution of various predictors to halophilic protein prediction, we quantified the importance of the descriptors by iteratively eliminating single descriptors from the three models and comparing the difference in AUROC values and AUPRC values. These results indicated that using all descriptors to obtain features might not be the best approach for all models. For catBoost, tri‐peptide composition (TPC) and dipeptide deviation from expected (DDE) feature descriptors had a negative impact on prediction (Figure 2A and Table S1). For XGBoost, composition of K‐spaced amino acid pairs (CKSAAP), transition (CTDT), correlation of triplet amino acids (CTriad), DDE, dipeptide composition (DPC), and grouped dipeptide composition (GDPC) feature descriptors had a negative impact on prediction (Figure 2B and Table S2). For RF, composition (CTDC), CTDT, CTirad, DDE, and TPC had a negative impact on the prediction (Figure 2C and Table S3).

Therefore, we eliminated descriptors with negative contributions and subsequently recoded using new descriptors for each model with a fivefold cross‐validation method. The results showed that after removing the respective descriptors, the performance of the catBoost model was improved (Table S1); also, the training speed was significantly increased and the calculation time was reduced. Similarly, the performance of XGBoost and RF had also been improved accordingly. Therefore, we confirmed that the model with the best features was the best model based on the fivefold cross‐validation method.

**Figure 3:**
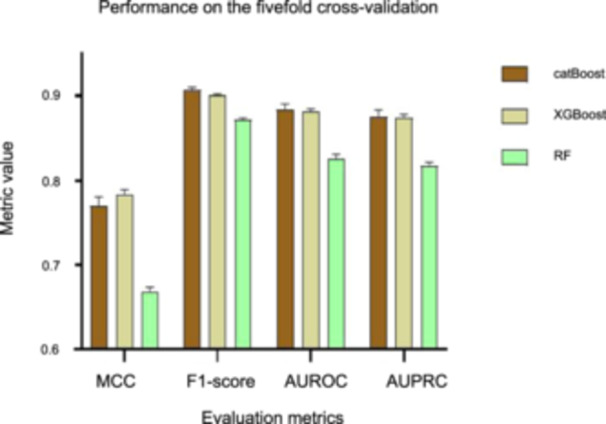
Performance comparison of three models in fivefold cross‐validation after removing descriptors with negative contributions. MCC, Matthew correlation coefficient.

Next, we compared the best performance of the three models, as shown in Figure 3. Obviously, catBoost generally outperformed the other models in all evaluation metrics. Moreover, the AUROC and AUPRC of catBoost exceeded 0.8845 and 0.8762, with a very small standard deviation of only 0.0062 and 0.0074, respectively (Table S1). This observation further indicated that catBoost not only showed high prediction performance but also high robustness. Therefore, the catBoost model and a combination of nine descriptors, including AAC, CKSAAP, CTDC, CTDT, distribution (CTDD), CTriad, DPC, grouped amino acid composition (GAAC), and GDPC, were retained for training the final model.

During the descriptor selection phase of the catBoost training data (Figure 2A), we observed that deleting one or two feature descriptors had minimal impact on the predictions of the catBoost model, with negligible AUROC and AUPRC fluctuations remaining below 0.01. This phenomenon could be attributed to three potential reasons: (1) Noise in certain features: Some features might introduce noise and make a small contribution or even have a negative impact on the prediction performance of the model. Removal of these features simplified the model, making it easier to train while maintaining high performance. (2) Robustness of the catBoost model: The catBoost model that we adopted shows strong generalization ability and could maintain high performance even when the number of features was reduced^13^. (3) Interactions between features: Although some features were less effective when used alone, they might have a significant impact when combined with other features during the training process. Therefore, it might not be sufficient to rely solely on the individual performance of features in the selection process. This could be explained by catBoost's excellent ability to handle high‐dimensional sparse data and feature selection. Removal of high‐dimensional feature descriptors (such as TPC with 8000 features) could notably enhance the performance of the model. This improvement was particularly significant for model performance, given that the initial training sample size exceeded 20,000 data points^18^.

Based on extensive research on halophilic proteins, it was found that AAC plays an important role in distinguishing halophilic and nonhalophilic proteins (2). The results of feature selection in this study further validated this observation. As shown in Figure 2, using descriptors such as AAC, CKSAAP, or DPC alone can produce effective predictive outcomes.

It must be acknowledged that the use of handcrafted features primarily based on AAC and some physicochemical properties to train the model may pose several challenges, such as the difficulty in distinguishing site‐directed mutations. This could significantly reduce the usefulness of the method in halophilic protein engineering. The focus of the study is to collect a rich dataset and train a highly accurate classifier model. In the future, we hope to use more comprehensive datasets and better algorithms, such as graph neural networks or pretrained large language models or even interpretable models, to accomplish the design task of halophilic proteins.

### Further optimization of the model for enhanced performance

In this study, we aimed to enhance the model performance of the catBoost model by using four feature selection methods, including chi‐square (Chi2), L1‐based feature selection, tree‐based feature selection, and variance threshold feature selection. The optimal feature selection method was determined based on the results obtained from fivefold cross‐validation and an independent test.

**Figure 4:**
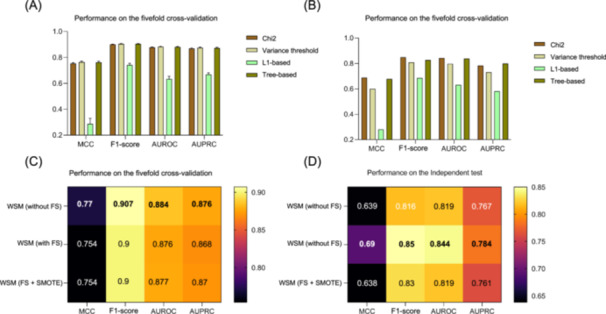
Performance evaluation of different optimization methods. (A) Performance evaluation of different feature selection methods on fivefold cross‐validation. (B) Performance evaluation of different feature selection methods on independent testing. (C) Performance comparison of catBoost without FS, with FS, and with FS + SMOTE on fivefold cross‐validation. (D) Performance comparison of catBoost with different processing methods on independent testing. FS, feature selection; SMOTE, synthetic minority oversampling technique; WSM, whole sequence model. The performance was evaluated based on the catBoost model with screened features.

Although the Chi2 feature selection method did not yield the highest Matthew correlation coefficient (MCC), F1‐score, AUROC, and AUPRC among the four feature selection methods (Figure 4A and Table S4), the model demonstrated a notably strong performance on the independent test set (Figure 4B and Table S5). This outcome indicated that the Chi2 feature selection method had better generalization ability.

We combined all handcrafted features encoded by eight descriptors with the catBoost model and evaluated the performance of the catBoost model on the fivefold cross‐validation (Table S6) and independent test (Table S7) by integrating the selected features using the Chi2 feature selection strategy. Although the performance of the model decreased slightly after using the feature selection method (Chi2) (Figure 4C), we could see improvements in MCC, F1 score, AUROC, and AUPRC on the independent test set (Figure 4D). This suggest that feature selection can indeed improve the generalization ability of the catBoost model.

Considering the data imbalance in the dataset, we attempted to solve this issue by using the synthetic minority oversampling technique (SMOTE) algorithm to balance the dataset and applying Chi2 feature selection to identify the best features. However, after introducing SMOTE into the model, it is evident from Figures 4C,D that a significantly darker color of the squares was observed compared to using feature selection alone, indicating that the performance of the model was significantly reduced based on fivefold cross‐validation and an independent test. Instead, we used catBoost with the Chi2 feature selection method as our final HPClas model. Despite the class imbalance, this model achieved strong predictive performance without using class balancing techniques such as SMOTE. In summary, feature selection provided a high‐quality model, while SMOTE oversampling performed poorly for HPClas.

To prove the superiority of the ML method that we provided, we further compared the HPClas model with the commonly used BLAST and HMMER methods on an independent test set. The results are listed in Table 2. Our ML method achieved an accuracy of 84.5% on a test set containing 100 positive and negative samples. In comparison, the accuracy rates of the other two methods were only 64.5% and 64.0%.

**Table 2:** Performance comparison of the three methods on independent test datasets.

Method	TP	TN	Accuracy (%)
HPClas	**88**	**81**	**84.5**
BLAST	59	70	64.5
HMMER	54	74	64.0

The values in bold indicate the best performance. HPClas, Halophilic Protein Classifier; TN, the number of true negatives (i.e., correctly predicted nonhalophilic proteins); TP, the number of true positives (i.e., correctly predicted halophilic proteins).

However, false negatives still exist, which may be due to the fact that the number of positive samples is much greater than the number of negative samples in the train set. Therefore, to meet the needs of different users, we have introduced an adjustable threshold in the prediction setting. Only when the predicted probability is above this threshold will the protein be classified as a halophilic protein. Based on this optimization, users can set thresholds according to their own needs, reduce the false‐positive rate, and improve the success rate and efficiency of wet laboratory experiments.

### Performance comparison with the SOTA method on a previous test set

To further evaluate the performance of our model, we compared HPClas with other state‐of‐the‐art halophilic protein prediction models on the same dataset in Table 3. The dataset came from the study of Zhang et al.^8^, ^9^, consisting of 15 pairs of halophilic and nonhalophilic proteins. Additionally, we have confirmed that these 30 proteins did not appear in our previous training dataset. We used the HPClas model to predict labels for all proteins in the test set and compared the results with those reported in previous studies. Experimental results indicated that the HPClas model predicted both halophilic proteins and nonhalophilic proteins with an accuracy of 100%. In comparison, previous studies utilizing artificial neural networks (ANNs) and support vector machines (SVMs) achieved accuracies of 80% and 73%, respectively^8^, ^9^. These findings demonstrated that the HPClas model outperformed previous models in predicting both halophilic and nonhalophilic proteins and showing higher accuracy.

**Table 3:** Performance comparison of HPClas and previous studies on the previous testing dataset.

PDB ID	Actual type	Predicted type	
		HPClas	Previous studies
1DOI:A	HP	HP	HP
1NWZ:A	HP	HP	**NHP**
1TJO:A	HP	HP	HP
2B5W:A	HP	HP	HP
2CC6:A	HP	HP	HP
3IBM:A	HP	HP	HP
1ITK:A	HP	HP	HP
2AZ3:A	HP	HP	HP
2J5K:A	HP	HP	HP
1CNO:A	HP	HP	**NHP**
1NML:A	HP	HP	**NHP**
2VPN:A	HP	HP	HP
3IFV:A	HP	HP	HP
3IGN:A	HP	HP	HP
3BSM:A	HP	HP	HP
1FXA:A	NHP	NHP	NHP
1MZU:A	NHP	NHP	**HP**
2VXX:A	NHP	NHP	NHP
2CD9:A	NHP	NHP	NHP
2V18:A	NHP	NHP	NHP
3KGZ:A	NHP	NHP	**HP**
2FXG:A	NHP	NHP	**HP**
3B54:A	NHP	NHP	NHP
1Y6J:A	NHP	NHP	NHP
1ETP:A	NHP	NHP	NHP
3HQ6:A	NHP	NHP	NHP
3FXB:A	NHP	NHP	NHP
1RWZ:A	NHP	NHP	NHP
3I5C:A	NHP	NHP	NHP
2QJJ:A	NHP	NHP	**HP**

The bold indicates incorrect predictions. HP, halophilic protein; NHP, nonhalophilic protein.

In previous studies, the researchers initially collected positive and negative samples from two bacterial strains, *S. ruber* DSM 13855 and *P. luteolum* DSM 2379, to form a training set. They used AAC as a feature representation method and used ANN and SVM to achieve the optimal prediction performance. However, none of them could accurately predict the label of PDB IDs 1NWZ and 1CNO, which were photoreceptor PYP (photoactive yellow protein)^19^ and cytochrome c552^20^, respectively. One potential reason was that these proteins were membrane proteins with different AACs compared with other proteins. This disparity might lead to inaccurate predictions from previous models. In comparison, our model successfully identified these proteins and accurately predicted them, demonstrating the comprehensiveness of our data sources and the accuracy of the trained model.

To make our work more accessible, we developed the model as a local standalone tool and published it publicly on GitHub. Additionally, to meet different needs, we incorporated multiple flexible threshold options into the local standalone version of HPClas. Users can adjust these thresholds to prioritize precision or recall in predictions. By sharing the HPClas model code and providing adjustable threshold options, we emphasized the transparency and reproducibility of our approach and aimed to promote collaboration and communication between the academic and bioinformatics communities. We believe that this open and flexible approach should facilitate further advances in the research and discovery of halophilic proteins.

### Performance of HPClas model on real halophilic proteins

To evaluate the practical utility of HPClas in a real‐world setting, we tested 16 experimentally validated halophilic enzymes as shown in Table 4. We retrieved their amino acid sequences from the RCSB Protein Data Bank (PDB) database based on the accession codes provided. These sequences were used as queries against the trained HPClas model to predict whether they would be classified as halophilic or nonhalophilic.

**Table 4:** Performance of HPClas in experimental data.

Halophilic enzyme	Source	PDB number	Optimal and tolerable salt concentration	Predicted type	Reference
Cellulase	*Bacillus* sp. BG‐CS10	5EOC	Optimal 2.5 mol/l NaCl	HP	[21]
Carbonic anlydrase	Bovine	4CNR	Tolerable 3.0 mol/l NaCl	HP	[22]
Carbonic anlydrase	*Dunaliella*	1Y7W	Tolerable 2.0 mol/l NaCl	HP	[23]
Carbonic anlydrase	*Photobacterium profundum*	5HPJ	Optimal 0.5 mol/l NaCl	**NHP**	[24]
Alkaline phosphatase	*Halomonas* sp. 593	3WBH	Tolerable 1.0–4.0 mol/l NaCl	**NHP**	[25]
Malate dehydrogenase	*Salinibacter ruber*	4CL3		HP	[26]
Malate dehydrogenase	*Haloarcula marismortui*	4JCO		HP	[Not published]
Rnase H1	*Halobacterium salinarum*	4NYN	Tolerable 3.0 mol/l NaCl	HP	[Not published]
Nucleoside diphosphate kinase	*Haloarlula quadrata*	2ZUA	Tolerable 0.2–4.0 mol/l NaCl	HP	[27]
Nucleoside diphosphate kinase	*Halomonas* sp. 593	3VGS	Tolerable 2.0 mol/l NaCl	HP	[28]
Malate synthase	*Haloferax volcanii*	3OYX	Tolerable 3.0 mol/l KCl	HP	[29]
Endonuclease	*Aliivibrio salmonicida*	2PU3	Optimal 0.4 mol/l NaCl	**NHP**	[30]
α‐Amylase	*Halothermothnx orenii*	3BC9	Optimal 0.9 mol/l NaCl	HP	[31]
*α*‐Amylase	*Halothermothnx orenii*	1WZA	Tolerable 4.7 mol/l NaCl	HP	[3]
DHFR	*Haloferax volcanii*	2ITH	Tolerable 3.5 mol/l NaCl	HP	[32]

The bold indicates incorrect predictions.

Of the 16 known halophilic proteins, HPClas correctly predicted 13 of them as halophilic and three of them as nonhalophilic. The results demonstrated that HPClas could effectively (albeit imperfectly) identify true halophilic proteins in many cases. There were still some false‐negative predictions of which known halophilic proteins were misclassified. Nonetheless, predictions of true halophilic proteins proved that HPClas had learned relevant patterns and could generalize beyond the training data to identify halophilic proteins in practice. Future testing on a more diverse dataset of halophilic proteins could further validate the practical applicability of this model.

### Feature importance analysis

Importance analysis using the SHapley additive exPlanations (SHAP) method allowed us to better understand the decision‐making process of the model and the contributions of each feature to the prediction results^33^. This enabled us to make more targeted adjustments in feature selection and model optimization, improving the performance and robustness of the model. In addition, importance analysis could help us identify potential issues, such as redundant features and interrelationships between features, which need to be focused on during model development^34^.

**Figure 5:**
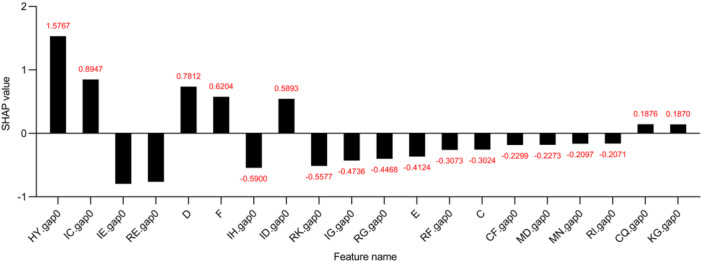
Feature importance analysis. It displays the statistical scores for each feature of the input obtained through SHAP (SHapley additive exPlanations) analysis. “XY.gap0,” where X and Y represent two different amino acids, typically denotes a count of zero‐gap amino acid pairs that shows variability within a protein sequence. In “*X*”, *X* represents a certain type of amino acid, and this feature indicates the frequency of occurrence of the amino acid represented by *X* in the entire protein.

In addition, this study conducted a coarse‐grained analysis of the model's prediction process. We evaluated the importance of each handcrafted input feature for classification and partial results are presented in Figure 5. Through the chart, we could clearly observe the crucial role of dipeptide combinations and the frequency of certain amino acids in classification decisions. Regarding the single amino acid content, it is evident that the presence of aspartic acid (D) significantly affected positive prediction, while glutamic acid (E) had a substantial impact on negative prediction. These findings were consistent with a 2014 statistical study of halophilic proteins by Giuseppe Graziano and colleagues^2^.

As for dipeptide‐type features, it is noteworthy that dipeptide features dominated the top‐ranking positions. Over the past three decades, protein dipeptide composition has played an important role in predicting ion channels^35^, protein structural categories^36^, subcellular localization^37^, nuclear receptors^38^, protein thermal stability^39^, nonclassical protein secretion^16^, protein secondary structure content^40^, and so forth. In this study, although a direct presentation of dipeptide feature descriptors was not provided, the identification of dipeptide features within the CKSAAP descriptor through feature selection indirectly highlighted the importance of these features in the field of halophilic protein prediction.

The interpretability analysis results of this study were consistent with the reported properties of halophilic proteins. Although we could not directly deduce all the characteristics of halophilic proteins and directly explain the identification process based on biological features, the results obtained were sufficient to prove that during the prediction process, the model did not rely on the “memory” training set, but truly learn halophilic‐related features and make predictions based on these features. This further confirmed the reliability of the model.

Through fine‐grained interpretability analysis of the model's prediction process, we gained insights into how the model made predictions and the features on which its decision depended. This not only enhanced our understanding of the model prediction results but also provided clues and inspiration for further biological research. We believe that this study will make valuable contributions to the identification and application of halophilic proteins.

## MATERIALS AND METHODS

### Collection of halophilic protein data

#### Collection of training sets

To obtain halophilic protein sequences, we searched the NCBI protein database^41^ using the keywords “halophilic bacteria” and “halophilic archaea”. Subsequently, SignalP 6.0 software was used to predict signal peptides for the resulting proteins^42^, and those lacking signal peptides were excluded. Then, The TMHMM^43^ online tool was used to predict the transmembrane regions, thereby excluding proteins containing transmembrane domains. The remaining protein, which contains a signal peptide and lacks a transmembrane region, was identified as a halophilic protein. Similarly, proteins with signal peptides from freshwater microorganisms were considered as nonhalophilic proteins. The collected redundant proteins were removed using CD‐HIT^44^ with a sequence identity threshold of 40%. Also, the protein length was restricted to between 150 and 4000 amino acids. These data collection and processing steps resulted in two diverse and comprehensive datasets, including halophilic and nonhalophilic proteins.

#### Collection of independent test sets

In terms of the test dataset, we primarily used the keywords “Halophilic bacteria” to search for halophilic proteins from UniProtKB and collected them as potential halophilic proteins. Then, the protein sequences of *Chryseobacterium* NBC 122 were collected from UniProtKB^45^ as a nonhalophilic sample because NBC 122 isolated from fresh water show optimal growth conditions in 1% NaCl and stop growing above 3.5% NaCl^46^. All sequences were pretreated by several steps, including redundancy reduction, length restriction, signal peptide prediction, and transmembrane region prediction. MMseqs. 2^47^ was used concurrently to ensure that the homology of samples in the test and training sets remained below 25%.

### Feature engineering

#### Feature descriptors

The 11 feature descriptors used include (1) AAC: This descriptor counts the frequency of each amino acid in a protein sequence; (2) CKSAAP: It counts the frequency of amino acid pairs with a specific spacing (K) between them; (3) DPC: This descriptor examines the frequency of consecutive dipeptides in a protein sequence; (4) TPC: It counts the frequency of amino acid tripeptides in the sequence; (5) CTDC: This descriptor characterizes the composition of a protein sequence based on three physicochemical groups; (6) CTDT: The CTDT descriptor measures the percentage frequency of amino acids following a specific amino acid belonging to a different physicochemical group; (7) CTDD: It describes the distribution of each physicochemical group along the protein sequence; (8) CTriad: This descriptor maps protein sequence into a frequency matrix based on amino acid triplets; (9) GAAC: It calculates the frequency of amino acids based on predefined amino acid groups; (10) GDPC: This descriptor calculates the frequency of dipeptides based on predefined amino acid groups; and (11) DDE: It examines the deviation between the observed frequency of dipeptides and the expected values in the protein sequence. All feature descriptors used here could be calculated using feature evolution and ML tools such as iFeature^48^, iLearn^49^, and iLearnPlus^50^. A detailed description of each feature descriptor is provided in the Supporting Information.

#### Feature normalization

For normalizing the values of different features, MinMaxScaler was used from the scikit‐learn package^51^. The normalization function could be formulated as follows:

$$x^{\prime }=\frac{x-x_{\min}}{x_{\max}-x_{\min}},$$

where x, $x_{\min}$, and $x_{\max}$ denote the original value, minimum value, and maximum value in the feature vectors, respectively, and $x^{\prime }$ denotes the scaled feature.

#### Feature selection

Several feature selection methods were used to construct the model, including chi‐square (Chi2)^52^, L1‐based feature selection^53^, tree‐based feature selection^54^, and variance threshold feature selection^55^. Chi2 feature selection is a univariate method that selects the best k features based on the chi‐square statistic. The L1‐based feature uses a linear model and L1 normalization penalty to eliminate features with zero coefficients^53^. Tree‐based feature selection calculates importance based on impurities and discards irrelevant features^54^. Variance threshold is a simple procedure based on feature variance that discards features below a certain variance threshold^55^. All four feature selection methods could be implemented using the “feature_selection” module in scikit‐learn.

### Model construction and optimization

#### Model construction

catBoost and XGBoost represent gradient‐boosting tree models, while RF is a tree‐based ensemble model^15^. During the training process of the first two models, performance enhancement was achieved by iteratively training a series of decision trees. In contrast, the RF model constructed multiple independently trained decision trees and made predictions through collective voting^56^.

When building these models, Python libraries associated with these three models were used. Initially, a classifier pipeline was built, including data preprocessing steps such as feature scaling (MinMaxScaler), feature selection (SelectKBest), and oversampling (SMOTE), as well as the classifiers themselves. In the classifier section, various parameters of each classifier were configured, including the number of iterations, learning rate, tree depth, and regularization parameters. Subsequently, the models were fitted using the training data, resulting in a trained classifier. Detailed model information is documented in the Supporting Information, and the code and specific hyperparameter values used for model training are publicly available in trainModel.py at https://github.com/Showmake2/HPClas.

#### The SMOTE

SMOTE^57^ was used to treat the class imbalance in our datasets. SMOTE operates by selecting samples from the minority class, identifying their nearest neighbors, and generating synthetic similar samples to increase the number of minority cases. This oversampling is implemented in the “imblearn” Python package.

#### Randomized fivefold cross‐validation

The benchmark dataset was randomly divided into five equal subsets. The evaluation process was repeated five times, with each subset used once as testing data and four times for training. In each iteration of cross‐validation, one subset was reserved for testing, while the remaining four subsets were combined into a training set for training the classifier. The results of the five tests were then averaged to obtain a single performance value that represents the overall performance of the classification model. Additionally, hyperparameter tuning was conducted using a grid search approach^58^. Scikit‐learn's KFold method^59^ was then used to implement fivefold cross‐validation, and the performance metrics of the model were presented as mean ± standard deviation.

#### Performance assessment

The seven performance indicators commonly used to evaluate model performance include precision, recall, accuracy, MCC^60^, F1 score, area under the receiver operating characteristic curve (AUROC), and area under the precision–recall curve (AUPRC). These performance metrics can be calculated as follows:

$$\mathrm{Precision}=\frac{\mathrm{TP}}{\mathrm{TP}+\mathrm{FP}},$$

$$\mathrm{Recall}=\frac{\mathrm{TP}}{\mathrm{TP}+\mathrm{FN}},$$

$$\mathrm{Accuracy}=\frac{\mathrm{TP}+\mathrm{TN}}{\mathrm{TP}+\mathrm{TN}+\mathrm{FP}+\mathrm{FN}},$$

$$\mathrm{MCC}=\frac{\mathrm{TP}*\mathrm{TN}-\mathrm{FP}*\mathrm{FN}}{\sqrt{\left(\mathrm{TP}+\mathrm{FP})(\mathrm{TP}+\mathrm{FN})(\mathrm{TN}+\mathrm{FP})(\mathrm{TN}+\mathrm{FN}\right)}},$$

$$F1‐\mathrm{score}=2\times \frac{\left(\mathrm{precision}\times \mathrm{recall}\right)}{\mathrm{precision}+\mathrm{recall}},$$

$$\mathrm{FPR}=\frac{\mathrm{FP}}{\mathrm{TN}+\mathrm{FP}},$$

where *TN*, *TP*, *FN*, and *FP* represent the number of true negatives (i.e., correctly predicted nonhalophilic proteins), true positives (i.e., correctly predicted halophilic proteins), false negatives (i.e., incorrectly predicted nonhalophilic proteins), and false positives (i.e., incorrectly predicted halophilic proteins), respectively. The *x*‐axis of the ROC curve represents the false‐positive rate (FPR) and the *y*‐axis represents the true‐positive rate (TPR). The precision–recall curve plots precision on the *y*‐axis and recall on the *x*‐axis. Consequently, the area under the precision–recall curve was also used as the primary metric to evaluate the performance of different models when the dataset is highly imbalanced. Since the dataset is imbalanced, the precision is insufficient to correctly evaluate the performance of the model; therefore, AUROC and AUPRC were used as the main evaluation matrix.

### Feature importance analysis

The SHAP value method was used to evaluate the importance score of each input feature predicted by the model^33^. Specifically, for a data point with N features, there exists N! possible ordering or permutations of those features. SHAP evaluates the marginal contribution of each feature by examining how the model output changes when a feature is added to an existing feature set. This is done for all possible feature permutations to account for interaction effects between features. The marginal contribution of a feature in each permutation was weighted by the probability of that permutation occurring. Finally, the SHAP value for each feature was computed as the weighted average of the marginal contributions of all permutations. In essence, the SHAP values explain how much each feature contributes to the output (either positively or negatively). This provides interpretability of how the model depends on specific input features.

## AUTHOR CONTRIBUTIONS

**Shantong Hu**: Data curation (equal); formal analysis (equal); investigation (equal); methodology (equal); software (equal); visualization (equal); writing—original draft (equal); writing—review and editing (equal). **Xiaoyu Wang**: Data curation (equal); supervision (equal); writing—review and editing (equal). **Zhikang Wang**: Formal analysis (supporting); visualization (supporting); writing—review and editing (equal). **Menghan Jiang**: Data collection. **Shihui Wang**: Supervision (equal). **Wenya Wang:** Funding acquisition (equal), project administration (equal). **Jiangning Song**: Methodology (supporting); supervision (equal); writing—review and editing (equal). **Guimin Zhang**: Conceptualization (lead); methodology (equal); project administration (equal); writing—review and editing (equal); funding acquisition (equal).

## ETHICS STATEMENT

The study in this article did not involve any trials on humans or animals.

## CONFLICT OF INTERESTS

The authors declare no conflict of interests.

## Supporting information

Supporting information.

## Data Availability

The code and dataset can be accessed at https://github.com/Showmake2/HPClas.
